# Fenestration after orthodontic traction of an impacted dilacerated maxillary central incisor: a case report and 4-year follow-up

**DOI:** 10.3389/fdmed.2026.1764953

**Published:** 2026-02-04

**Authors:** Shuhao Xu, Xiaolong Li, Yu Zhang, Wei Li

**Affiliations:** 1 Department of Stomatology, Deyang People’s Hospital, Deyang, China; 2 Department of Respiratory and Critical Care Medicine, Deyang People’s Hospital, Deyang, China

**Keywords:** case report, dilacerated teeth, fenestration, impacted teeth, orthodontic traction

## Abstract

Dilacerated impacted maxillary central incisors require early detection, early diagnosis, and early treatment. Early orthodontic traction can allow the root, which is confined by the buccal or palatal cortical bone, to rotate along with the crown and enter the cancellous bone, thereby gaining an opportunity for continued development. We reported a case of an 11-year-old boy with an impacted dilacerated maxillary central incisor. Following surgical exposure combined with orthodontic traction, fenestration occurred. However, after four years of follow-up, the affected tooth exhibited normal pulp vitality and no abnormal mobility. This case might offer an indication that even for dilacerated maxillary central incisors with short roots, or those that develop fenestration after traction, the long-term outcomes of traction treatment could be quite favorable.

## Introduction

A dilacerated tooth is a developmental anomaly characterized by deviation of the crown or root from the tooth's long axis, often presenting as a distinct angular bend between the crown and root (or part of the root) (1, 2). It is a type of dental morphological development abnormality. Dilacerated anterior teeth most commonly occur in the upper anterior region and can lead to dental misalignment, significantly affecting both aesthetics and oral function (3). Therefore, dilacerated impacted maxillary central incisors require early detection, early diagnosis, and early treatment. For impacted dilacerated central incisors, timely orthodontic traction is a highly effective treatment approach (4). The optimal timing for traction is during the early stages of root development, as this promotes normal eruption of the permanent tooth and preserves dental integrity. Previous studies have shown that early release of impaction through orthodontic traction facilitates root development and increases total root length (5). Early orthodontic traction can allow the root, which is confined by the buccal or palatal cortical bone, to rotate along with the crown and enter the cancellous bone, thereby gaining an opportunity for continued development.

However, orthodontic traction carries risks such as inappropriate anchorage design, incorrect traction position and direction, root resorption, ankylosis, alveolar bone dehiscence and/or fenestration, and compromised gingival aesthetics (6, 7). If traction is initiated too late, or if the dilaceration has already formed and the apical foramen has closed before or during traction, there is a higher risk of fenestration and root resorption after orthodontic treatment, which may potentially affect the long-term survival of the tooth (8). Alveolar bone fenestration (a window affecting the root surface but still surrounded by bone) is one of the most common alveolar defects (9, 10). Such defects often result in root exposure and gingival recession, which can compromise the stability of orthodontic treatment, lead to relapse, or even contribute to therapeutic failure, thereby presenting significant challenges in orthodontic management (11, 12).

In cases of labially impacted and dilacerated maxillary central incisors, the root development is constrained by the palatal cortical plate, ultimately leading to a labial root curvature. Consequently, orthodontic traction in such cases will inevitably result in the root perforating the alveolar bone, causing fenestration. However, according to current literature reports, even in cases of dilacerated maxillary central incisors with short roots or those developing fenestration post-traction, the long-term outcomes of orthodontic traction remain relatively favorable. This may be attributed to the establishment of effective physical interlocking between the dilacerated root and alveolar bone, enabling the crown to withstand normal masticatory forces (13, 14). But currently, there is no consensus on whether subsequent fenestration in such cases affects long-term tooth health and treatment stability. This case report showed an 11-year-old boy with a severely labially impacted, dilacerated right upper central incisor that had missed the optimal traction window. Following orthodontic traction, apical bone fenestration occurred. However, after 4 years of follow-up, the traction-treated dilacerated tooth remained healthy and stable.

## Case presentation

An 11-year-old boy presented to our department with a chief complaint of “unerupted right upper anterior tooth for over 4 years.” The patient was previously healthy with no significant systemic medical history. The parents recalled a history of severe caries in the primary upper anterior teeth and a past episode of gingival abscess in the upper anterior region. There was no history of drug allergies.

**Figure 1:**
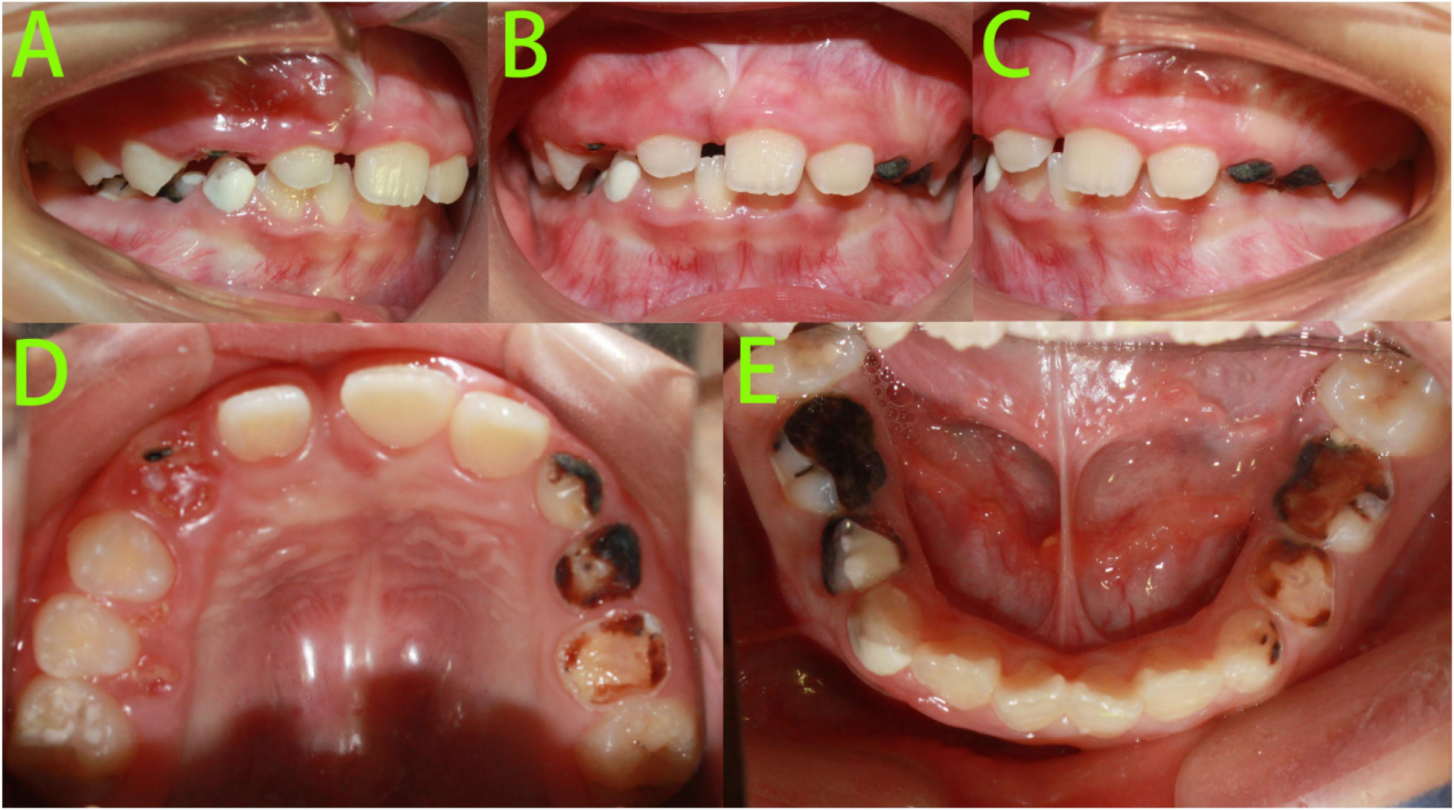
Pre-treatment intraoral photographs. **(A–E)** Intraoral photographs showed tooth 11 was not visible intraorally.

Intraoral examination (Figure 1) revealed: A mixed dentition stage with lateral teeth. Teeth 16, 14, 12, 21, 22, 26, 36, 32–42 had erupted and replaced their predecessors. Tooth 11 was not visible intraorally. Teeth 12 and 21 were tilted towards the edentulous space, with insufficient space for tooth 11. Tooth 53 presented as a residual root. Teeth 63–65, 73–75, and 83–85 exhibited occlusal wear and arrested caries.

**Figure 2:**
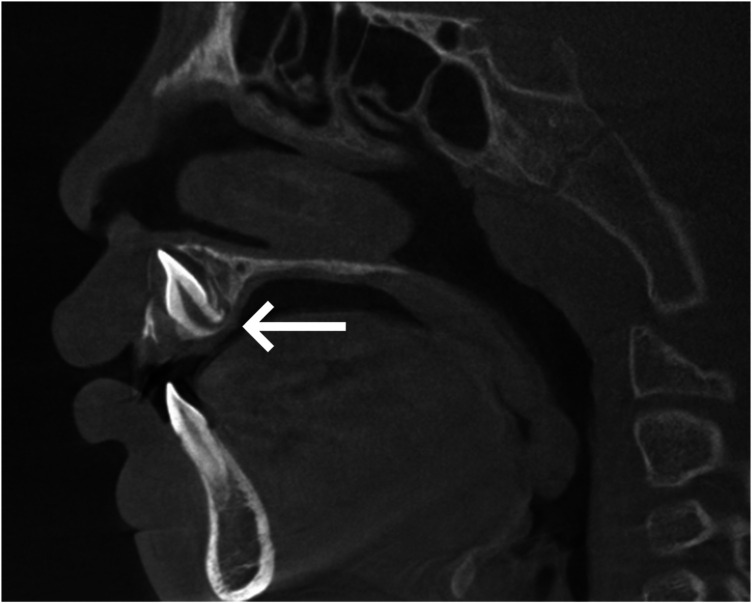
Pre-treatment CBCT. Tooth 11 was dilacerated and severely labially impacted, with a crown-root angulation of approximately 90 degrees (white arrow).

Pre-treatment cone-beam computed tomography (CBCT) (Figure 2) revealed: Tooth 11 was dilacerated and severely labially impacted, with a crown-root angulation of approximately 90 degrees.

Diagnosis: (1) dilacerated and impacted tooth 11, (2) residual root of tooth 53, (3) arrested caries on teeth 63–65, 73–75, and 83–85.

Based on a comprehensive evaluation, the following treatment plan was formulated: (1) oral hygiene instruction, (2) regular outpatient follow-up to monitor the exfoliation and replacement of carious primary teeth, (3) surgical exposure followed by orthodontic traction to align tooth 11. (The parents were specifically informed that due to the missed optimal treatment window and the severe crown-root angulation, the procedure carries significant risks, including tooth mobility, fenestration, and root resorption. The long-term prognosis of the affected tooth may be unfavorable.)

The patient's parents were thoroughly informed of the diagnosis and treatment plan, and treatment was initiated after obtaining their informed consent.

A 2 × 4 fixed partial appliance was bonded. A push-coil spring was used to create eruption space for tooth 11. Three months later, surgical exposure was performed to uncover the palatal surface of the tooth 11 crown, followed by bonding of a lingual button and initiation of orthodontic traction with light occlusally-directed forces to erupt tooth 11.

**Figure 3:**
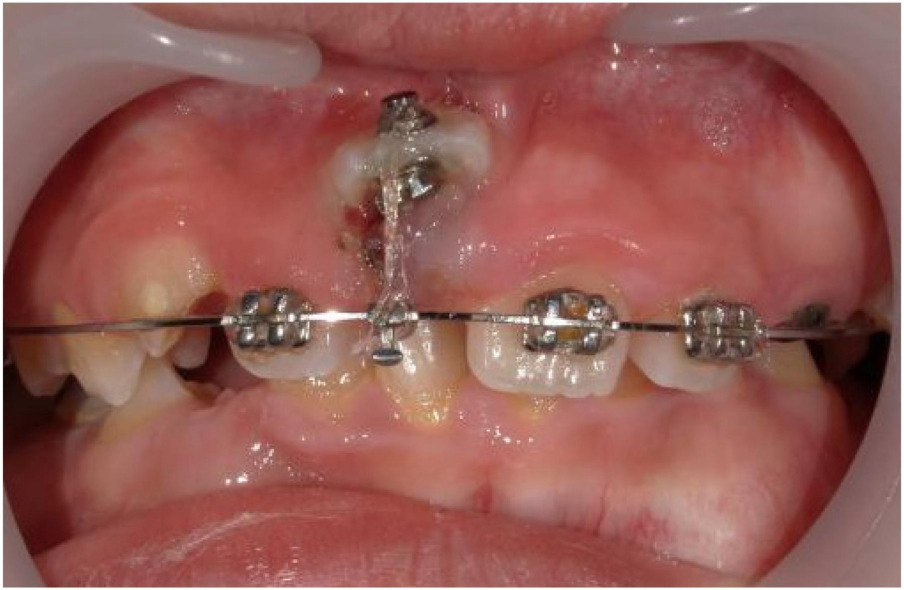
6-month intraoral photograph. Tooth 11 was effectively erupted with the crown visible in the oral cavity.

Six months into treatment (Figure 3), tooth 11 was effectively erupted with the crown visible in the oral cavity. A lingual button was re-bonded to the labial surface of the crown, and light occlusally-directed traction was continued.

**Figure 4:**
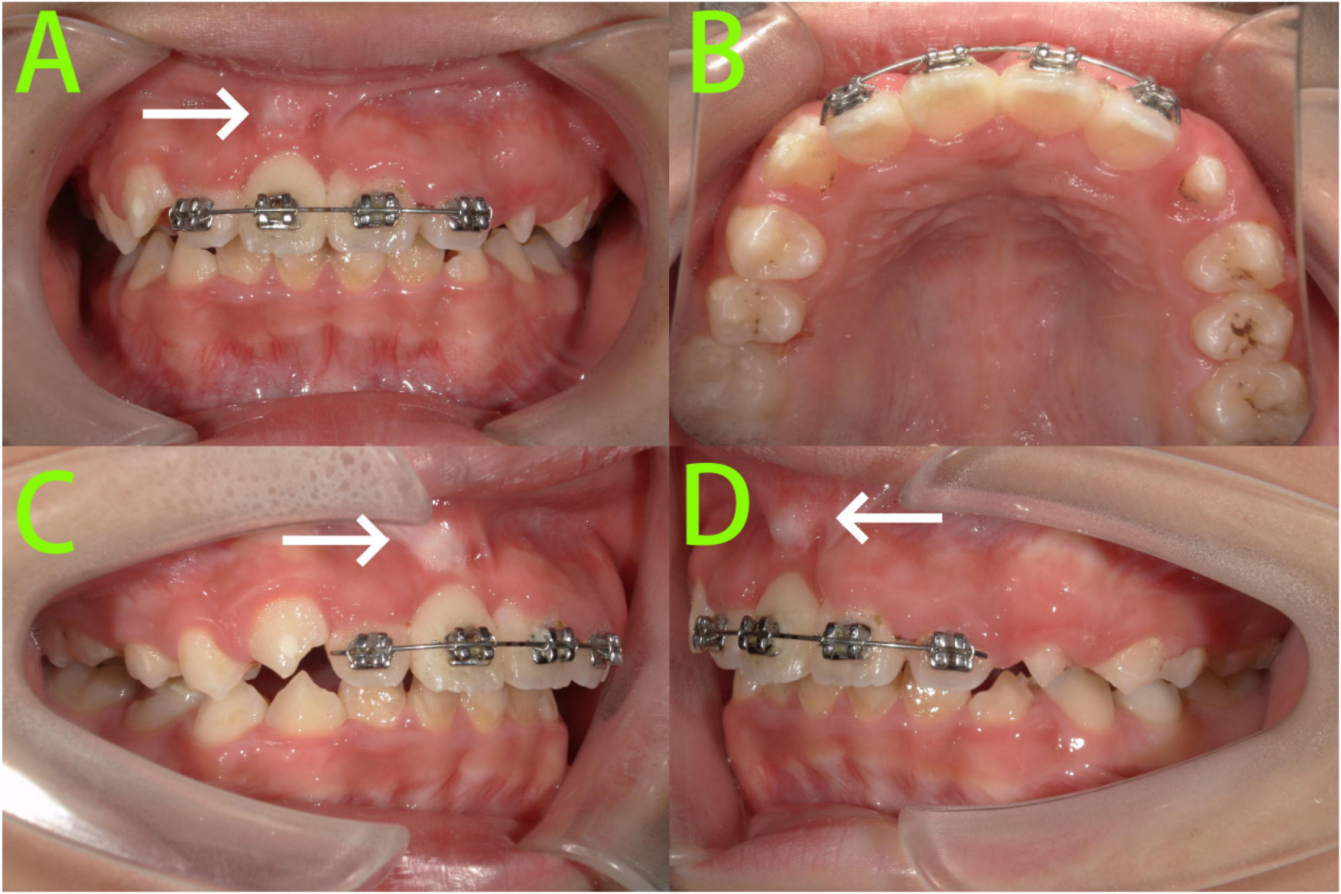
9-month intraoral photographs. **(A–D)** Intraoral photographs showed tooth 11 was effectively aligned through traction, and a prominent root contour was visible in the gingiva at the apical region on the labial aspect (white arrow).

After 9 months of treatment (Figure 4), tooth 11 was effectively aligned through traction. A prominent root contour was visible in the gingiva at the apical region on the labial aspect. A sectional archwire was placed on the upper anterior teeth for retention.

**Figure 5:**
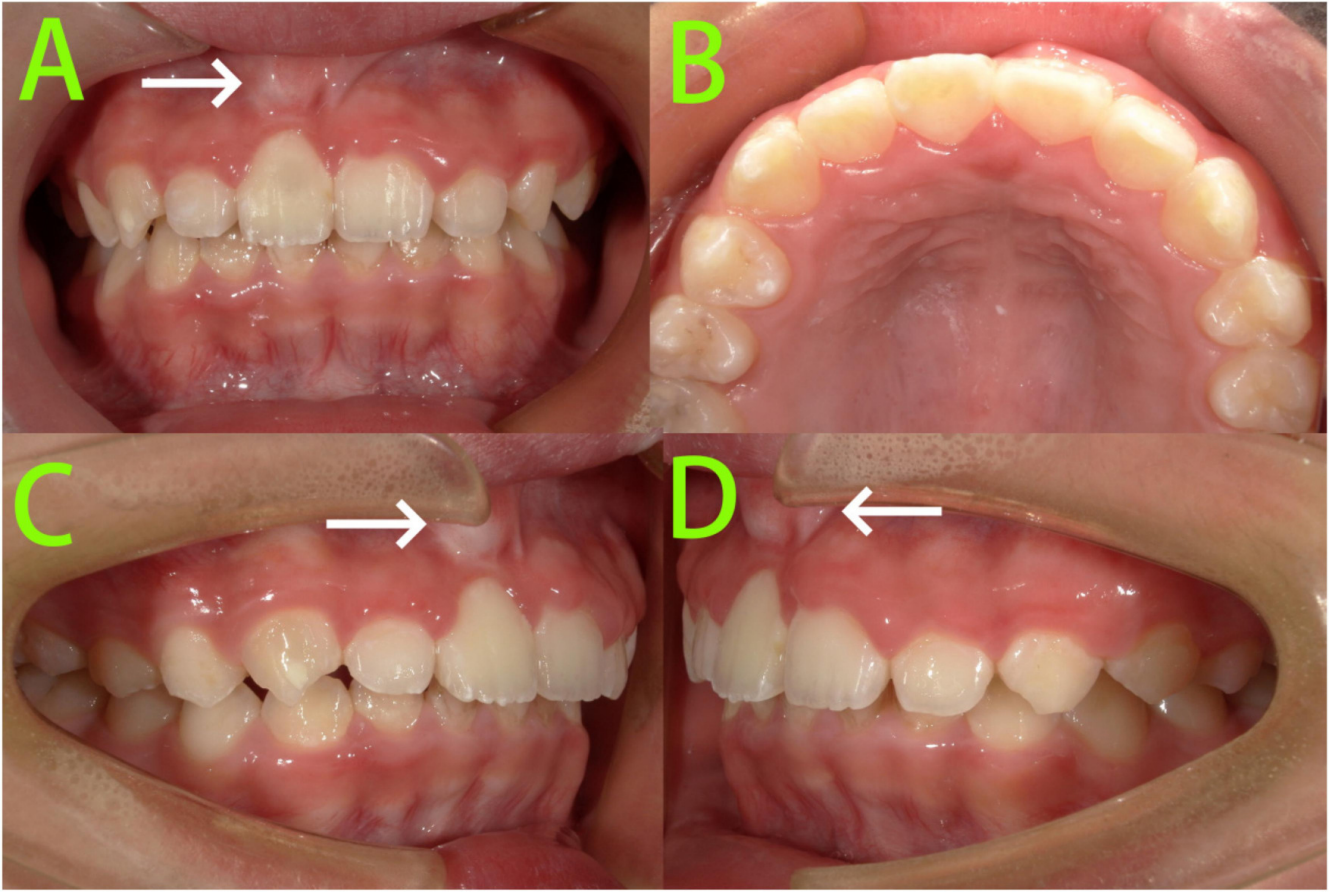
Post-treatment intraoral photographs. **(A–D)** Intraoral photographs showed tooth 11 was well-aligned, with a prominent root contour visible in the gingiva at the apical region on the labial aspect (white arrow).

After 15 months of treatment (Figure 5), the upper anterior fixed appliance was removed. Tooth 11 was well-aligned, with a prominent root contour visible in the gingiva at the apical region on the labial aspect. No significant mobility was observed, and pulp vitality testing showed no obvious abnormalities. The remaining primary teeth had exfoliated normally and were replaced by their permanent successors, resulting in a generally well-aligned full dentition. The anterior overjet was normal, with a slightly deep overbite.

**Figure 6:**
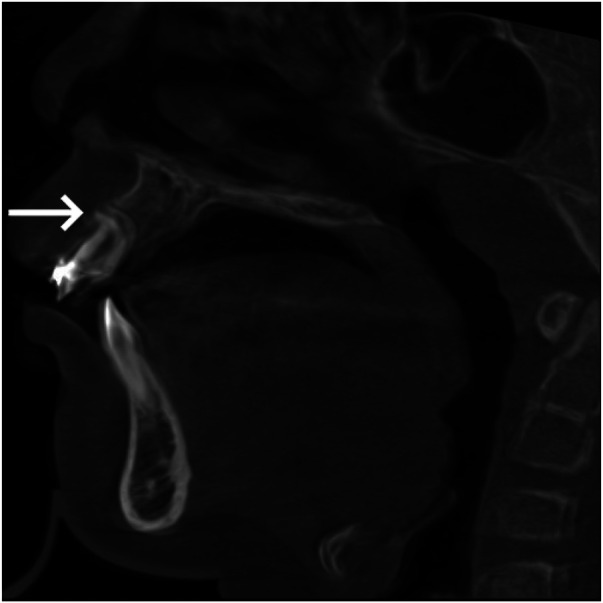
Post-treatment CBCT. Tooth 11 maintained a crown-root angulation of approximately 90 degrees, with radiographic evidence of fenestration in the apical region (white arrow).

A follow-up CBCT scan revealed (Figure 6) that tooth 11 maintained a crown-root angulation of approximately 90 degrees, with radiographic evidence of fenestration in the apical region.

Despite the occurrence of fenestration, tooth 11 maintained satisfactory aesthetics, health, function, and stability. The patient and parents expressed considerable satisfaction with the treatment outcome, even after being informed of the tooth's potentially unfavorable long-term prognosis. During the retention phase, nightly wear of the maxillary Hawley retainer was prescribed for one year, with the subsequent retention plan to be determined by follow-up evaluations.

**Figure 7:**
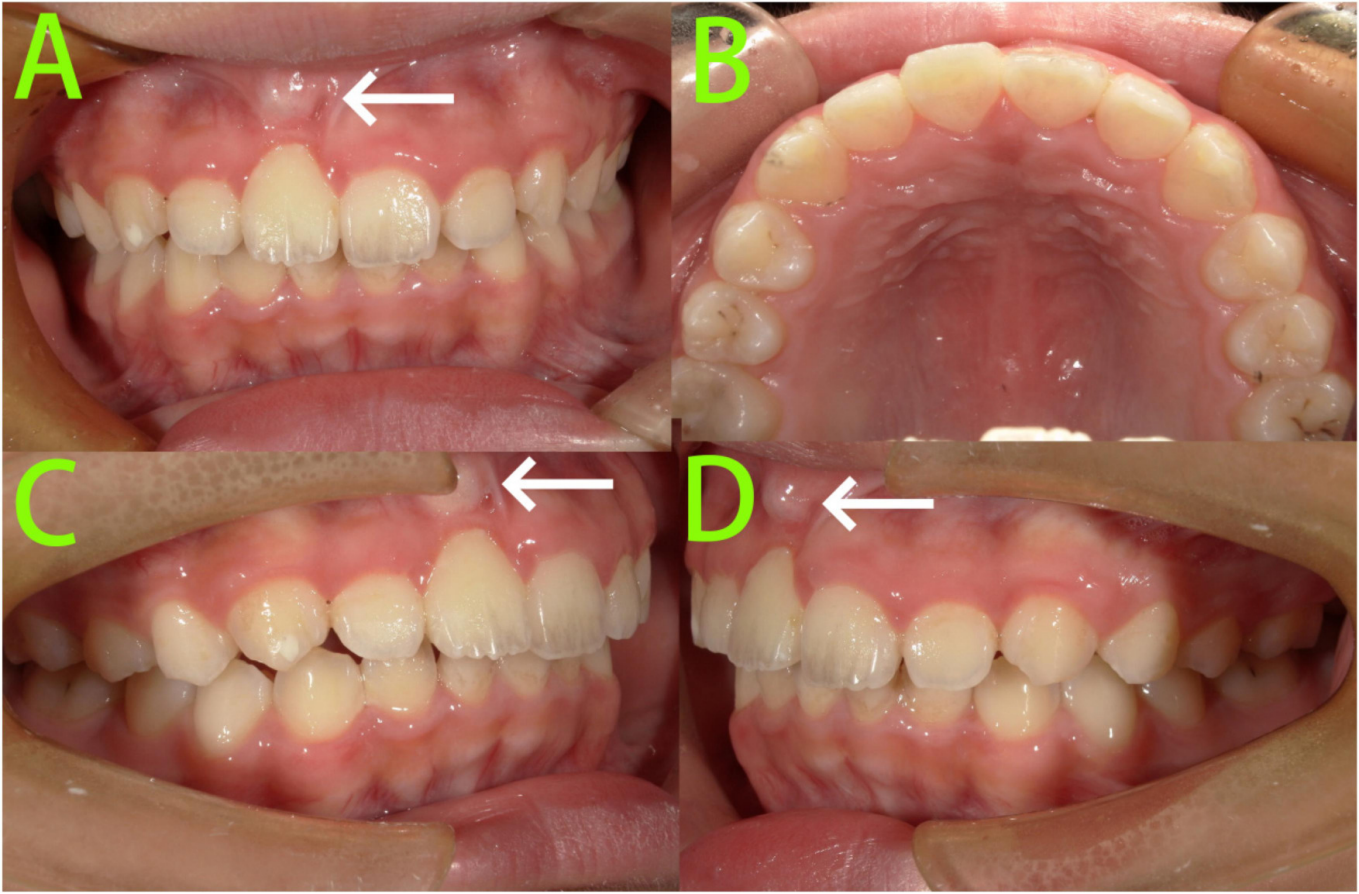
1-year follow-up intraoral photographs. **(A–D)** A prominent root contour remained visible in the gingiva at the apical region on the labial aspect of tooth 11 (white arrow).

At the 1-year retention follow-up (Figure 7), a prominent root contour remained visible in the gingiva at the apical region on the labial aspect of tooth 11. However, the tooth exhibited normal crown color, positive pulp vitality, and no significant pathological mobility, although slight labial relapse was noted. No premature occlusal contact was detected in the anterior region. Subsequently, the retention protocol was adjusted to wearing the maxillary Hawley retainer 2–3 nights per week.

**Figure 8:**
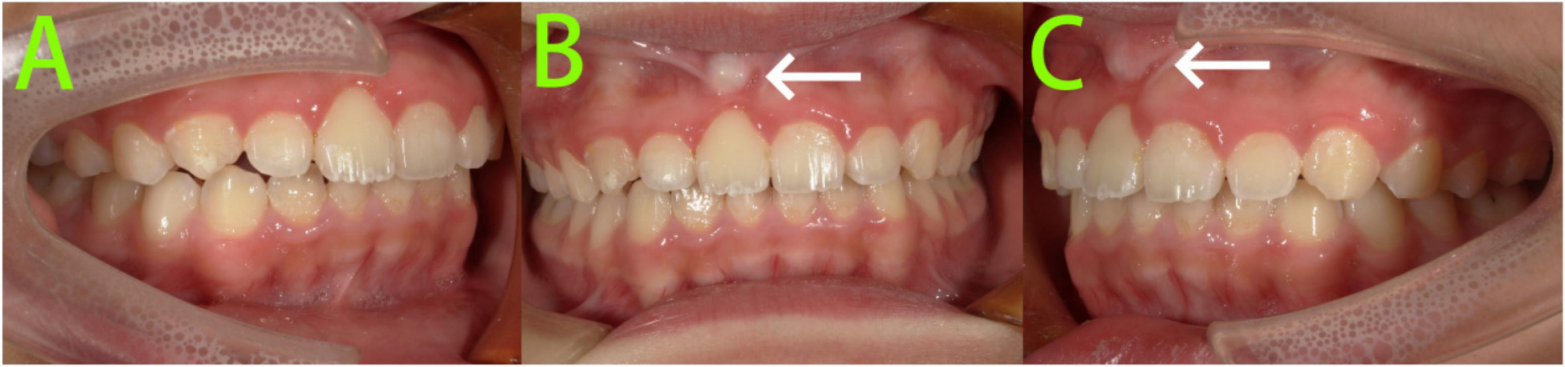
4-year follow-up intraoral photographs. **(A–C)** Tooth 11 exhibited slight labial inclination, and the root contour on the labial apical aspect appeared more prominent (white arrow).

At the 4-year retention follow-up (Figure 8), the patient reported no discomfort and normal functional use of the tooth. However, tooth 11 exhibited slight labial inclination, and the root contour on the labial apical aspect appeared more prominent. The tooth maintained normal pulp vitality and showed no significant pathological mobility. No premature occlusal contact was detected in the anterior region. The patient and parents expressed pleasant surprise at the long-term maintenance of the treatment outcome. It was noteworthy that although clinical examination revealed a slight labial relapse, neither the patient nor his parents perceived it, reporting no impact on aesthetics or masticatory function. Given the absence of patient complaints and the lack of significant abnormal findings on clinical examination, no additional imaging studies were performed. The patient was instructed to discontinue wearing the maxillary Hawley retainer. We will continue to monitor the case through regular follow-up appointments.

## Discussion

Impaction of the maxillary central incisors is the third most common type of impaction, with an incidence rate of approximately 0.03%–2.1% (15). Although impacted maxillary central incisors are relatively rare, they pose significant challenges for both patients and professionals. Due to the unique position of these teeth, their absence not only has a substantial impact on facial aesthetics but also affects function, phonetics, and psychological well-being (16). Impacted dilacerated maxillary central incisors represent an even more challenging scenario among cases of impacted maxillary central incisors. Impacted dilacerated anterior maxillary teeth are usually caused by acute mechanical trauma to the primary anterior teeth (17, 18) or developmental interfering factors, including apical periodontitis of primary teeth, cleft lip and palate, ectopic tooth germ development, soft tissue scarring, insufficient space or interference from surrounding structures, odontomas, dental follicles, ankylosed primary teeth, genetic factors, and certain syndromes, among others (19). In this case, the patient had a history of severe caries in the primary anterior teeth during early childhood and experienced gingival abscesses in the upper anterior teeth. These factors likely contributed to the dilaceration and severe labial impaction of the maxillary central incisor. This further underscores the importance of primary teeth health for the proper eruption of permanent teeth, highlighting the significant and ongoing task of preventing and managing caries in primary teeth.

The diagnosis of impacted dilacerated maxillary incisors is typically based on clinical and radiographic findings. Clinical signs of impacted dilacerated teeth include retained primary teeth, insufficient space in the area of the unerupted tooth, and abnormal elevation of the palatal or labial mucosal soft tissues. Preoperative determination of the morphology and position of the unerupted tooth is a critical factor in diagnosis and treatment planning. Therefore, radiographic evaluation, especially CBCT, is essential for confirming the presence of impaction, identifying the location and orientation of the impacted tooth, and assessing its relationship with adjacent structures (20). Additionally, CBCT aids in assessing the thickness of the cortical and cancellous bone, thereby assisting dentists in selecting the optimal treatment plan and preventing complications (21).

The treatment of impacted dilacerated incisors requires a multidisciplinary approach, involving two primary treatment methods: extraction of the affected tooth followed by prosthetic restoration, or successful management of the impacted incisors through surgical exposure followed by orthodontic traction (22). The success of surgical exposure and orthodontic traction in treating impacted dilacerated incisors primarily depends on the maturity of the tooth root, the position and angulation of the impacted tooth, and the availability of sufficient space (23). The most common complications associated with orthodontic traction for impacted dilacerated incisors include fenestration, ankylosis, attachment loss, external root resorption, pulp necrosis, pulpal obliteration, crown discoloration, root exposure, and unaesthetic gingival margins (24). Therefore, the selection of a treatment plan for impacted dilacerated incisors requires careful consideration.

The formation of the root is primarily regulated by the combined actions of Hertwig's epithelial root sheath and the dental follicle. When factors such as trauma or periapical infection of the primary tooth cause the forming root to deviate at an angle from the crown, the subsequent growth of the root continues under the coordinated regulation of the epithelial root sheath and dental follicle, progressing along a direction that deviates from the normal long axis of the root, thereby resulting in the formation of a curved root, known as dilaceration (25). Early orthodontic traction of a dilacerated tooth can help move its root away from the cortical bone and into the cancellous bone, thereby gaining more space for growth. Under the influence of orthodontic traction, the epithelial root sheath, once separated from the cortical bone, can induce a secondary curve, guiding the root to continue growing along the long axis of the tooth (19). Therefore, early orthodontic traction for impacted dilacerated teeth that have not yet developed root curvature can reduce the incidence of dilacerated root formation. For teeth that have already developed dilaceration, early orthodontic traction can utilize the developmental potential of the epithelial root sheath to induce a secondary curve, guiding continued root development. This process helps increase root length and reduce the crown-root angulation (26, 27). Therefore, in selecting the treatment plan for this case with impacted dilacerated maxillary incisor, surgical exposure combined with orthodontic traction was ultimately chosen.

In orthodontic traction of impacted dilacerated incisors, successful outcomes are often more likely in cases where the tooth has a younger dental age, an open apex, a more horizontally inclined and inverted crown, a lower tooth position, and an obtuse crown-root angulation (28). In this case, the patient had an older dental age, a severe inverted crown angulation, a high tooth position, and a crown-root angulation of approximately 90 °, all of which are unfavorable factors for orthodontic traction. Although the affected tooth was successfully repositioned through traction in the later stages, fenestration occurred in the apical region. Healthy alveolar bone and periodontal support are crucial for tooth protection. Fenestration may compromise the health and stability of the tooth, leading to complications such as gingival recession (29). To prevent fenestration resulting from orthodontic treatment, careful treatment planning is essential to avoid displacing teeth beyond the alveolar bone boundaries (30). Currently, techniques such as periodontally accelerated osteogenic orthodontics (PAOO) have demonstrated promising results in the treatment of orthodontically induced fenestration (31). In this case, labial gingival recession eventually occurred, accompanied by noticeable apical bulging in the gingival area of the labial root apex. However, after four years of follow-up, it was highly encouraging that the affected tooth exhibited normal pulp vitality and no abnormal mobility. This case might offer an indication that even for dilacerated maxillary central incisors with short roots, or those that develop fenestration after traction, the long-term outcomes of traction treatment could be quite favorable. This might be attributed to the formation of a well-integrated physical interlock between the curved root and the alveolar bone, allowing the crown to withstand normal occlusal forces (13, 14). In addition to physical interlock, a stable functional occlusion is equally crucial for the long-term stability of impacted dilacerated incisors following traction.

During the retention phase, a slight labial relapse of the dilacerated central incisor was observed. However, no premature occlusal contact was detected in the anterior region during the clinical examination. Therefore, we considered that this slight relapse might be associated with two factors. First, a slight labial inclination may allow the root to be positioned more within the cancellous bone, which could represent a more physiologically stable state. Additionally, the Hawley retainer used during the retention phase provided relatively poor control over anterior crown torque, which may have contributed to the slight labial relapse.

While numerous reports exist on the treatment of impacted dilacerated incisors, there remains a lack of studies focusing on long-term treatment stability, particularly in cases complicated by fenestration. This gap underscores the unique contribution of the present case report. Although this case provided some clinical insights, it still had limitations. First, during the surgical exposure, adopting a closed-eruption method rather than simple gingivectomy might have helped preserve or enhance labial gingival attachment (32). Additionally, after the completion of traction, performing root resection could potentially position the root in a more stable location within the cancellous bone (8). Of course, continued follow-up was necessary for this case to determine the long-term stability of dilacerated teeth accompanied by fenestration. Finally, this remained only a single case report, and future clinical studies are needed for further validation.

## Conclusion

For impacted dilacerated maxillary central incisors, early detection, diagnosis, and treatment are essential. Early orthodontic traction can help relocate the root away from the cortical bone and into the cancellous bone, providing space for growth. Additionally, it fully leverages the inductive potential of the epithelial root sheath to generate a secondary curvature, guiding continued root development, thereby increasing root length and reducing the crown-root angulation. This case report suggested that even for dilacerated maxillary central incisors with short roots, or those that develop fenestration after traction, the long-term outcomes of traction treatment might still be favorable. Additionally, orthodontic traction could prevent alveolar bone resorption and adjacent tooth tipping that typically follow tooth loss. Even if the tooth became mobile over time due to inadequate occlusal load, the procedure could help preserve sufficient alveolar bone volume for future implant restoration in adulthood. Future clinical studies are still needed for further validation.

## Data Availability

The raw data supporting the conclusions of this article will be made available by the authors, without undue reservation.
